# Identification of a Prognostic Signature Model with Tumor Microenvironment for predicting Disease-free Survival after Radical Prostatectomy

**DOI:** 10.7150/jca.51173

**Published:** 2021-02-22

**Authors:** Hao Zhao, Xuening Zhang, Zhan Shi, Bingxin Guo, Wenli Zhang, Kun He, Xueqi Hu, Songhe Shi

**Affiliations:** 1 Department of Epidemiology and Biostatistics, College of Public Health, Zhengzhou University, Zhengzhou 450001, China.; 2 Department of Medicine, Zhengzhou First People's Hospital, Zhengzhou 450004, China.; 3 Department of Urology, Henan Province Hospital of Traditional Chinese Medicine, Zhengzhou 450002, China.

**Keywords:** prostate cancer, radical prostatectomy, microenvironment, stromal, immune, prognosis, prediction

## Abstract

**Background:** The tumor microenvironment (TME) and immune checkpoint inhibitors have been shown to promote active immune responses through different mechanisms. We attempted to identify the important prognostic genes and prognostic characteristics related to TME in prostate cancer (PCa).

**Methods:** The gene transcriptome profiles and clinical information of PCa patients were obtained from The Cancer Genome Atlas (TCGA) database, and the immune and stromal scores were calculated by the ESTIMATE algorithm. We evaluated the prognostic value of the risk score (RS) model based on univariate Cox analysis and least absolute shrinkage and selection operation (LASSO) Cox regression analysis and established a nomogram to predict disease-free survival (DFS) in PCa patients. The GSE70768 dataset was utilized for external validation. Twenty-two subsets of tumor-infiltrating immune cells were analyzed using the CIBERSORT algorithm.

**Results:** In this study, the patients with higher immune/stromal scores were associated with a worse DFS, higher Gleason score, and higher pathological T stage. Based on the immune and stromal scores, 515 differentially expressed genes (DEGs) were identified. The univariate Cox and LASSO Cox regression models were employed to select 18 DEGs from 515 DEGs and construct an RS model. The DFS of the high-RS group was significantly lower than that of the low-RS group (*P*<0.001). The AUCs for the 1-year, 3-year and 5-year DFS rates in the RS model were 0.890, 0.877 and 0.841, respectively. A nomogram of DFS was established based on the RS and Gleason score, and the AUCs for the 1-year, 3-year and 5-year DFS rates in the nomogram were 0.907, 0.893, and 0.872, respectively. These results were further validated in the GSE70768 dataset. In addition, the proportion of Tregs was determined to be higher in high-RS patients (*P*<0.05), and the expression levels of five immune checkpoints (CTLA-4, PD-1, LAG-3, TIM-3 and TIGIT) were observed to be higher in high-RS patients (*P*<0.05).

**Conclusions:** Our study established and validated an 18-gene prognostic signature model associated with TME, which might serve as a prognosis stratification tool to predict DFS in PCa patients after radical prostatectomy.

## Introduction

Prostate cancer (PCa) is the most common malignant tumor among men in Western world 1. According to the 2018 Global Cancer Report, the incidence of prostate cancer among men is second only to lung cancer, and the incidence is gradually increasing, with an annual growth rate as high as 8.92% 2, 3. With the progress of cancer treatment technology, localized PCa can be cured by radical prostatectomy (RP), and mortality is also significantly reduced. However, the recurrence rate of PCa after RP is still high, resulting in treatment failure. Previous studies have reported that approximately 40% of patients relapse within five years after RP, and approximately 27-53% of patients eventually develop local recurrence or distant metastasis within 10 years after RP 4, 5.

In recent years, immunotherapy with cytokines and immune checkpoint inhibitors has been shown to promote active immune responses through different mechanisms 6. Therefore, a new classification of PCa in combination with immunotherapy is needed to more accurately predict postoperative recurrence, thereby contributing to clinical decision making to achieve personalized treatment and reduce the recurrence rate among PCa patients.

The tumor microenvironment (TME) refers to the surrounding microenvironment of tumor cells, including immune cells, stromal cells, endothelial cells, inflammatory cells, and fibroblasts 7. Among these cells, tumor-infiltrating immune cells and stromal cells are two major non-tumor cell components, which have been considered important for the diagnosis and prognostic evaluation of cancer patients 8. Therefore, understanding the cell composition and function of the TME has considerable potential to effective prevent cancer recurrence and promote immunotherapy responses.

Bioinformatics analysis uses a combination of biological, statistical, computer science, and informatics techniques to process and analyze large amounts of complex biological data 9. The establishment of public databases, such as The Cancer Genome Atlas (TCGA) database and Gene Expression Omnibus (GEO) database, provides new data resources and technical means for TME research 10, 11. Yoshihara et al 12 first proposed the ESTIMATE (Estimation of Stromal and Immune cells in Malignant Tumor tissues using Expression data) algorithm in 2013. This algorithm uses the unique properties of the transcription profile of cancer samples to infer infiltrating stromal/immune cells. According to reports, researchers have explored the tumor characteristics and prognostic assessment of lung cancer 13, breast cancer 14, and clear-cell renal cell carcinoma 15 based on the ESTIMATE algorithm. However, the value of immune and stromal scores for PCa has not been verified to data.

In this study, we examined the TME in PCa patients, calculated the immune and stromal scores for each cancer sample, and established a risk score (RS) prognostic model and a nomogram combining the RS and Gleason score using the TCGA database. Moreover, these results were validated using the GEO database. Finally, based on the CIBERSORT (Cell type Identification by Estimating Relative Subpopulations of RNA Transcription) method, we explored the relationship between high-RS and low-RS PCa patients and immune cell infiltration and immune checkpoints to provide a foundation for future efforts to achieve precise immunotherapy and postoperative management of PCa patients.

## Materials and Methods

### Data collection and processing

We obtained the fragments per kilobase million (FPKM) data of RNA-Seq from the TCGA-PRAD cohort (https://portal.gdc.cancer.gov/), including 499 PCa patients and 52 normal samples. Next, the FPKM data was transferred to transcripts per million (TPM) expression data. The gene expression levels of duplicate samples were averaged, and normal samples were deleted for subsequent analysis.

We used the Genomic Data Commons (GDC) tool and cBiopPortal website (http://www.cbioportal.org/) to download the corresponding clinical information, including age, pathological T stage, lymph node status, Gleason score, surgical margin status, tumor laterality, and prognostic information. We excluded samples with incomplete key clinical information, and finally included 480 PCa patients for the following analysis. We utilized the “limma” package for normalization processing. Next, immune, stromal and ESTIMATE scores were calculated using the ESTIMATE algorithm. For GEO database, the inclusion criteria were as follows: (1) patients diagnosed with PCa; (2) patients who had undergone RP; and (3) patients with detected gene levels in tissue samples. The exclusion criteria were as follows: (1) clinical data without prognostic information and (2) dataset with a small sample size (n<50). Finally, the eligible dataset, GSE70768 (n=111), was selected, and the normalized expression matrix was used for subsequent analysis.

### Correlation analysis and survival analysis

For data satisfying the parameter tests, the *t*-test was utilized for comparisons between two groups, and ANOVA was employed for comparisons of three groups or more. For test data with the unsatisfactory parameters, the Wilcoxon test was used for comparisons between two groups, and the Kruskal-Wallis analysis was used for comparisons of three groups or more. The relationship between the immune/stromal score and important clinical phenotypes was explored by comparing the differences in the immune/stromal score in different clinical subgroups. Disease-free survival (DFS), as the main prognostic endpoint, was defined as the time from the date of diagnosis to the date of recurrence or death and the last follow-up.

According to the stromal/immune score of each PCa patient, the best cut-off value based on the R package “maxstat” (i.e., the maximum selective rank statistic method) was used to divide the patients into high and low score groups 16. Based on “survival” packages, the difference in DFS between the two groups was evaluated by the Kaplan-Meier (K-M) method and log-rank test.

### Differentially expressed gene (DEG) screening

The “limma” package in R software was used to screen for DEGs between the high and low groups of immune/stromal scores. In this study, an adjusted *P* value <0.05 and a fold change ≥1.5 were regarded as the critical values for screening DEGs. The immune-related DEGs and stromal-related DEGs showing the same expression trend were selected using a Venn diagram. We used the “ggplot2” and “pheatmap” packages to generate a volcano plot and heatmap.

### DEG functional enrichment analysis

The David online database (http://david.ncifcrf.gov) was used to explore the potential functions of DEGs. Gene ontology (GO) analysis included biological processes (BPs), molecular functions (MFs) and cellular components (CCs), which were demonstrated by bar plots. The Kyoto Encyclopedia of Genes and Genomes (KEGG) was used to conduct the pathway analysis, which was illustrated by a dotplot. With a false discovery rate (FDR) <0.05 as the cut-off value, all enrichment results were visualized with the “ggplot2” package.

### Establishment of a prognostic signature model and survival analysis

A univariate Cox model was used to determine the relationship between TME-related DEG expression and DFS. Next, least absolute shrinkage and selection operator (LASSO) Cox regression analysis was used to develop an optimal risk signature with the minimum number of genes 17. A set of genes and their coefficients were determined by the minimum criteria, which involved selecting the best penalty parameter λ associated with the 10-fold cross validation 18. The RS was calculated as follows: RS = ∑ (βi * Expi) ('i' = the number of prognostic hub genes, 'βi' represents the coefficient of each gene, and 'Expi' represents gene expression.) In addition, PCa patients were divided into high-RS and low-RS groups according to the optimal cut-off value of the risk score. A receiver operating characteristic (ROC) curve was then used to assess the predictive ability of the RS model. The K-M method and log-rank test were used to analyze the differences in survival between the high-RS group and the low-RS group.

### Validation of the prognostic signature model in the test dataset

The GSE70768 independent dataset was used for verification. A scatter plot was used to show the distribution of gene expression profiles and the RS, and the Pearson correlation coefficient was used calculate the correlation. Immunohistochemistry (IHC) images of the selected prognosis-related genes in high- and low-grade tissue were retrieved from the Human Protein Atlas online database (http://www.proteinatlas.org). According to the RS calculation formula of the training dataset, the samples in the test dataset were divided into the high-RS group and the low-RS group. K-M survival analysis and ROC curve analysis were used to evaluate the predictive ability of this model.

### Identification of independent prognostic factors

Univariate and multivariate Cox analysis were used to study the independent prognostic value of the RS and other clinical characteristics, and a clinical prediction model was established using a nomogram. Next, the performance of the nomogram was evaluated by time-dependent ROC curve analysis.

### Estimating the composition of immune cells

CIBERSORT is a deconvolution algorithm based on the principle of linear support vector regression used to describe the infiltration of immune cells in the sample. LM22 is composed of 547 genes that accurately distinguish 22 human hematopoietic cell phenotypes, including seven T cell types, naïve and memory B cells, plasma cells, NK cells, and myeloid subsets 19. We utilized CIBERSORT and LM22 to jointly estimate the scores of 22 human immune cell types in PCa samples from the TCGA cohort. For each sample, the sum of all estimated immune cell type scores was equal to 1. We compared differences in the composition of immune cell types between high-RS and low-RS groups.

### Statistical analysis

Statistical analysis was performed using R software (version 3.6.1). All statistical tests were two-sided, and a *P* value <0.05 was considered to be significant.

## Results

### Immune score and stromal score were correlated with clinical features of PCa

The workflow chart of this study is shown in **Figure S1**. A total of 480 male PCa patients were included in the TCGA database. Elderly PCa patients (≥65 years) accounted for 33.54%. There were 186 patients (38.75%) with ≤pT2c stage disease, 153 patients (31.88%) with pT3a stage disease, and 141 patients (29.38%) with ≥pT3b stage disease. Regarding the Gleason score, 44 cases (9.17%) were in the <7 score group, 240 cases (50%) were in the 7 score group, and 196 cases (40.83%) were in the >7 score group. For other detailed information, see **Table 1** for clinical information.

**Figure 1:**
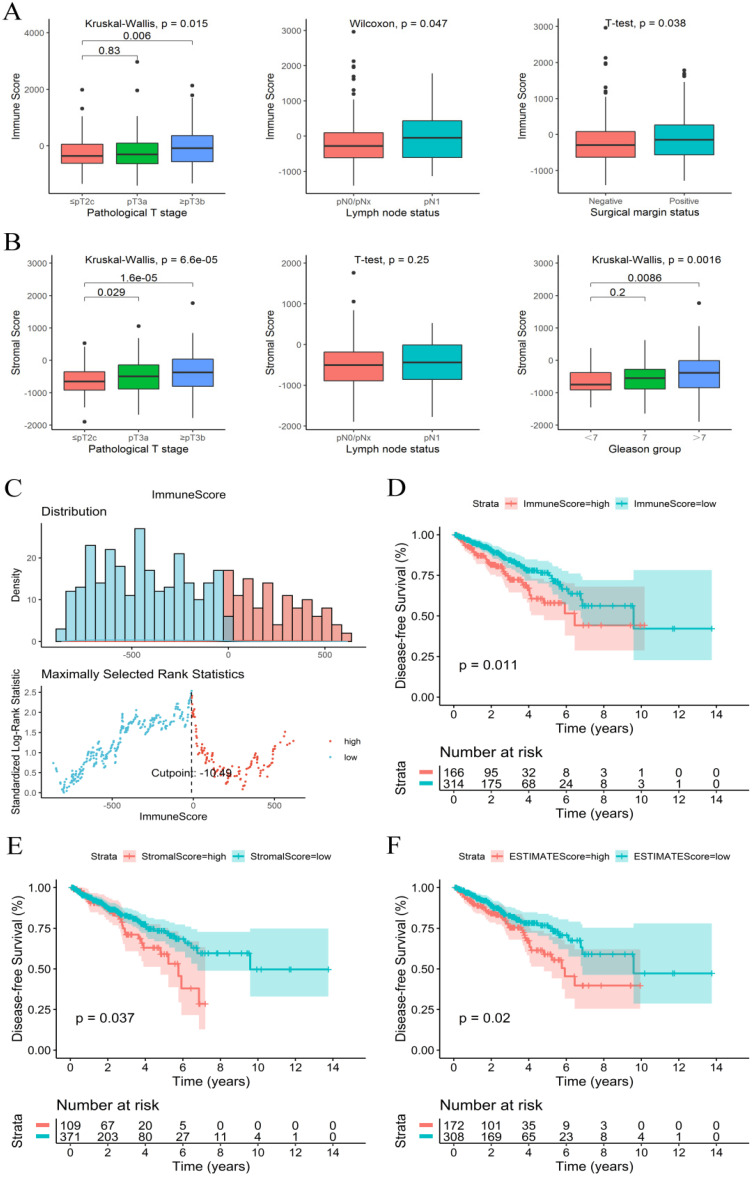
Immune score and stromal score were correlated with clinical features and prognosis of PCa. **(A)** Immune score correlated with clinical features. **(B)** Stromal score correlated with clinical features. **(C)** The best cut-off value for the immune score. **(D-F)** K-M survival curves of the relationship between the immune, stromal and ESTIMATE scores and PCa patient prognosis.

The immune, stromal and ESTIMATE scores of each sample were calculated using an ESTIMATE algorithm. The immune score ranged from -1404.50 to 2963.33, the stromal score ranged from -1897.04 to 1762.53, and the ESTIMATE score ranged from -3237.41 to 3584.35. The relationship between immune and stromal scores and clinical characteristics showed that a higher immune score was significantly associated with a higher T stage (*P*=0.015), positive surgical marginal status (*P*=0.038) and lymph node metastasis (*P*=0.047) **(Figure 1A)**. A higher stromal score was significantly associated with a higher T stage (*P*<0.001) and higher Gleason score (*P*=0.002) **(Figure 1B)**. The relationship between the ESTIMATE score and clinical characteristics was similar to that of the stromal score **(Figure S2)**.

### Immune score and stromal score were significantly related to PCa prognosis

The K-M survival curves of the relationship between immune and stromal scores and PCa patient prognosis showed that patients with lower immune and stromal scores had higher DFS rates (*P*=0.011 and *P*=0.037, respectively) **(Figure 1D and 1E)**. PCa patients with low ESTIMATE scores also consistently had higher DFS rates than patients with high ESTIMATE scores (*P*=0.02) **(Figure 1F)**. These observations consistently suggested that patients with a low immune or stromal score had a more favorable outcome.

### Identification of DEGs based on the immune score and stromal score in PCa

**Figure 2:**
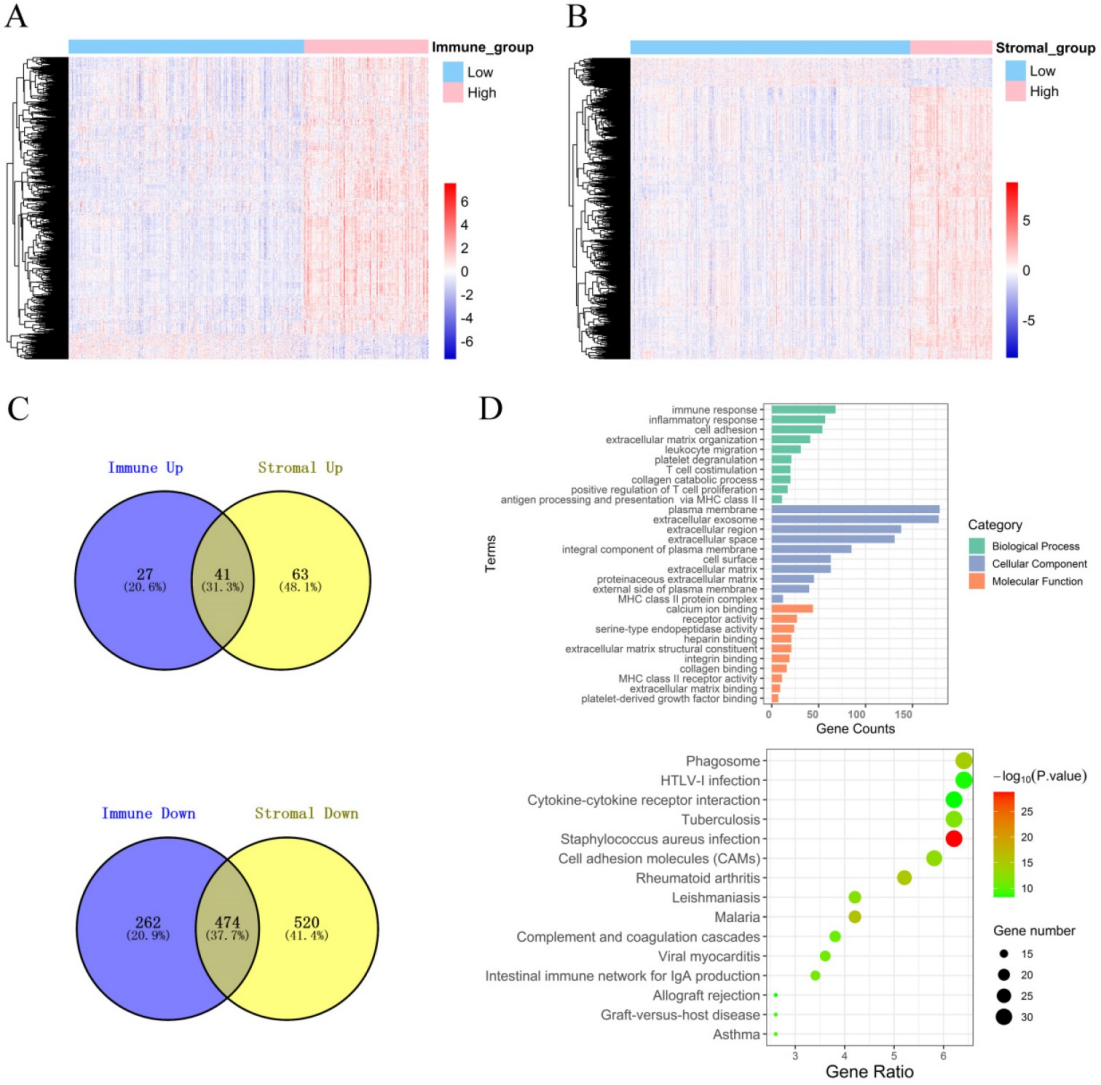
Identification of DEGs and function and pathway enrichment analysis. **(A)** A heatmap of 804 DEGs between patients with high or low immune scores. **(B)** A heatmap of 1098 DEGs between patients with high or low stromal scores. **(C)** Cross-up-regulated and cross-down-regulated DEGs between the immune and stromal groups. **(D)** Function and pathway enrichment analysis of DEGs by GO and KEGG.

To explore the DEGs that are closely related to the TME, the “limma” package was used to process the Affymetrix microarray data from 480 PCa patients. **Figure 2A** shows a heatmap of 804 DEGs between high and low immune scores, and **Figure 2B** shows a heatmap of 1098 DEGs between high and low stromal scores.

In addition, the volcano plot shows DEGs based on the immune score and stromal score **(Figure S3)**. For the immune score, there were 68 up-regulated DEGs and 736 down-regulated DEGs in the high group compared with the low group. For stromal score, compared with the low score group, there were 104 up-regulated DEGs and 994 down-regulated DEGs in the high score group. A Venn diagram showed 41 cross-up-regulated DEGs and 474 cross-down-regulated DEGs between the immune and stromal groups **(Figure 2C)**.

### Function and pathway enrichment analysis of DEGs

Functional enrichment analysis for DEGs, including BPs, CCs, MFs and KEGG pathways were conducted using the David gene annotation tool. BPs indicated that these genes may be associated with the immune response, inflammatory response, cell adhesion, extracellular matrix organization, and leukocyte migration. CCs indicated that these genes may be associated with the plasma membrane and extracellular exosome, region, and space. MFs indicated that these genes may be associated with calcium ion binding, receptor activity, and serine-type endopeptidase activity. The results of KEGG enrichment were related to the immune response, including phagosomes, infection, cytokine receptor interactions, and cell inflammatory molecules (CAMs) **(Figure 2D)**. Overall, our results confirmed that TME-related DEGs were closely related to the anti-tumor immunity of PCa patients.

### Establishment of a prognostic signature model and survival analysis

**Figure 3:**
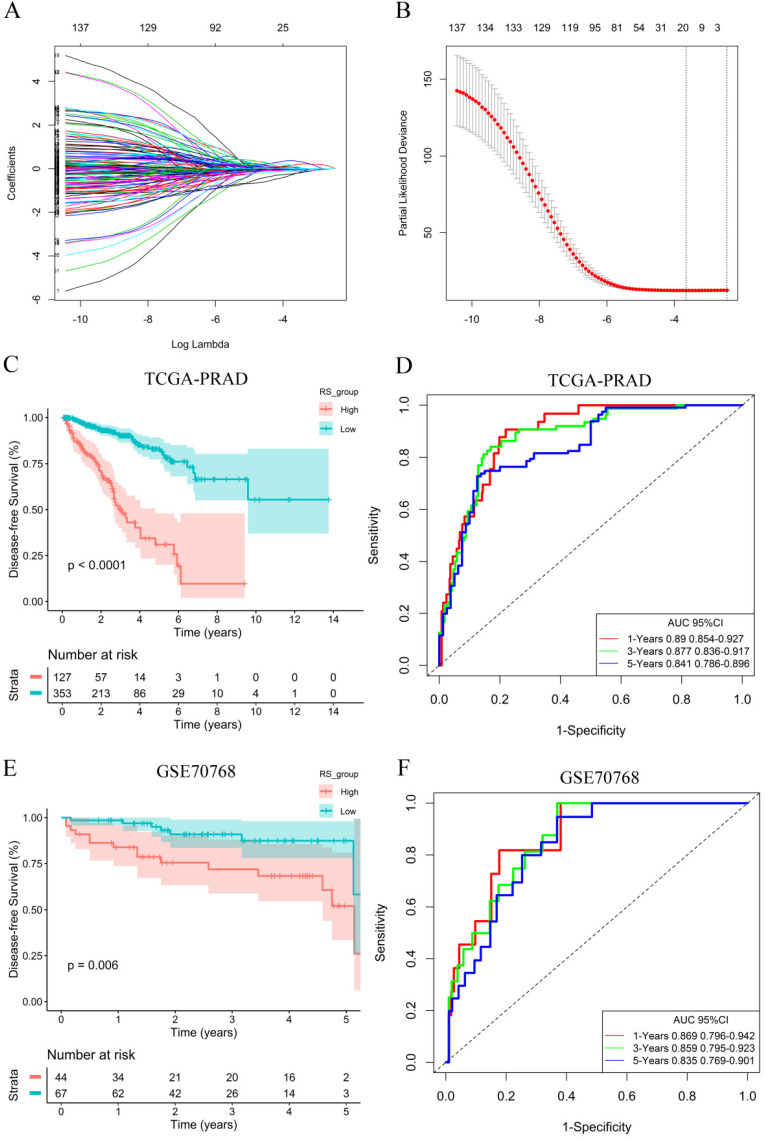
RS model calculation and survival analysis. **(A)** LASSO coefficient profiles. **(B)** The 10-fold cross-validation results that identified optimal values of the penalty parameter λ. **(C)** K-M curves of the high- and low-RS groups in the TCGA-PRAD dataset.** (D)** ROC curve based on the RS model in the TCGA-PRAD dataset. **(E)** K-M curves of the high- and low-RS groups in the GSE70768 dataset. **(F)** ROC curve based on the RS model in the GSE70768 dataset.

To explore the potential role of DEGs in DFS, a univariate Cox proportional hazards regression model was first developed, and the results showed that 172 prognostic genes were selected by univariate analysis. Next, the 10-fold cross-validation random sampling method was used, and according to the -2 log-likelihood test, through repeated calculation and verification, the model was optimized at the penalty parameter log *λ*= -1.59, and a risk score (RS) model of 18 genes was constructed **(Figure 3A, 3B)**. RS =(0.06410*C1QC) +(0.00467*COL1A1) + (0.01777*HOPX) +(0.17512*ITGAX) + (0.35768*STAB1) + (0.13676*TGFB1) + (-0.04815*APOF) + (-0.06180*CHRNA2) + (-0.22487*CLIC6) + (-0.03191*EGR1) + (-0.01361*FEV) + (-0.00269*FOS) + (-0.06470*GJB1) + (-0.23936*GNG2) + (-0.10059*HSD11B1) + (-0.03021*OLFML3) + (-0.06134*PLTP) + (-0.03940*TGM3) **(Figure S4)**. In addition, survival curves of 18 DEGs were constructed to explore the prognostic value of each gene **(Figures S5 and S6)**. These results of K-M curves showed that PCa patients with low expression levels of C1QC, COL1A1, HOPX, ITGAX, STAB1, and TGFB1 had a better prognosis. In contrast, we found that low expression of other hub genes was associated with a poor prognosis in PCa patients.

A total of 353 patients with risk scores below the critical value (-0.17539) were classified as the “low-RS group”, and the remaining 127 patients were classified as the “high-RS group” according to the optimal cut-off value of the RS. The K-M curve showed that the DFS of high-RS patients was significantly worse (*P*<0.001) **(Figure 3C)**. At the same time, for sensitivity verification, the RS was grouped by the median, and the results were consistent **(Figure S7A)**. To determine whether the RS can predict DFS without considering residual tumors, we performed a risk-stratified analysis. The results showed that in the absence of residual tumors, patients with a low RS also had significantly longer DFS than patients with a high RS (*P*<0.001) **(Figure S7B)**.

To evaluate the predictive ability of the RS model, we drew an ROC curve based on the RS and calculated the AUC. The AUCs for the 1-year, 3-year and 5-year DFS rates in the RS model were 0.890, 0.877 and 0.841, respectively **(Figure 3D)**, indicating that the RS model had good predictive accuracy. To further compare the accuracy of the RS model, a prediction model based on the Gleance score was established. The AUCs for the 1-year, 3-year and 5-year DFS rates in the Gleason score model were 0.704, 0.677 and 0.682, respectively **(Figure S7C)**, and we found that the RS model was more accurate for predicting prognosis than the Gleason score model in the TCGA database.

### Validation of the prognostic signature model

**Figure 4:**
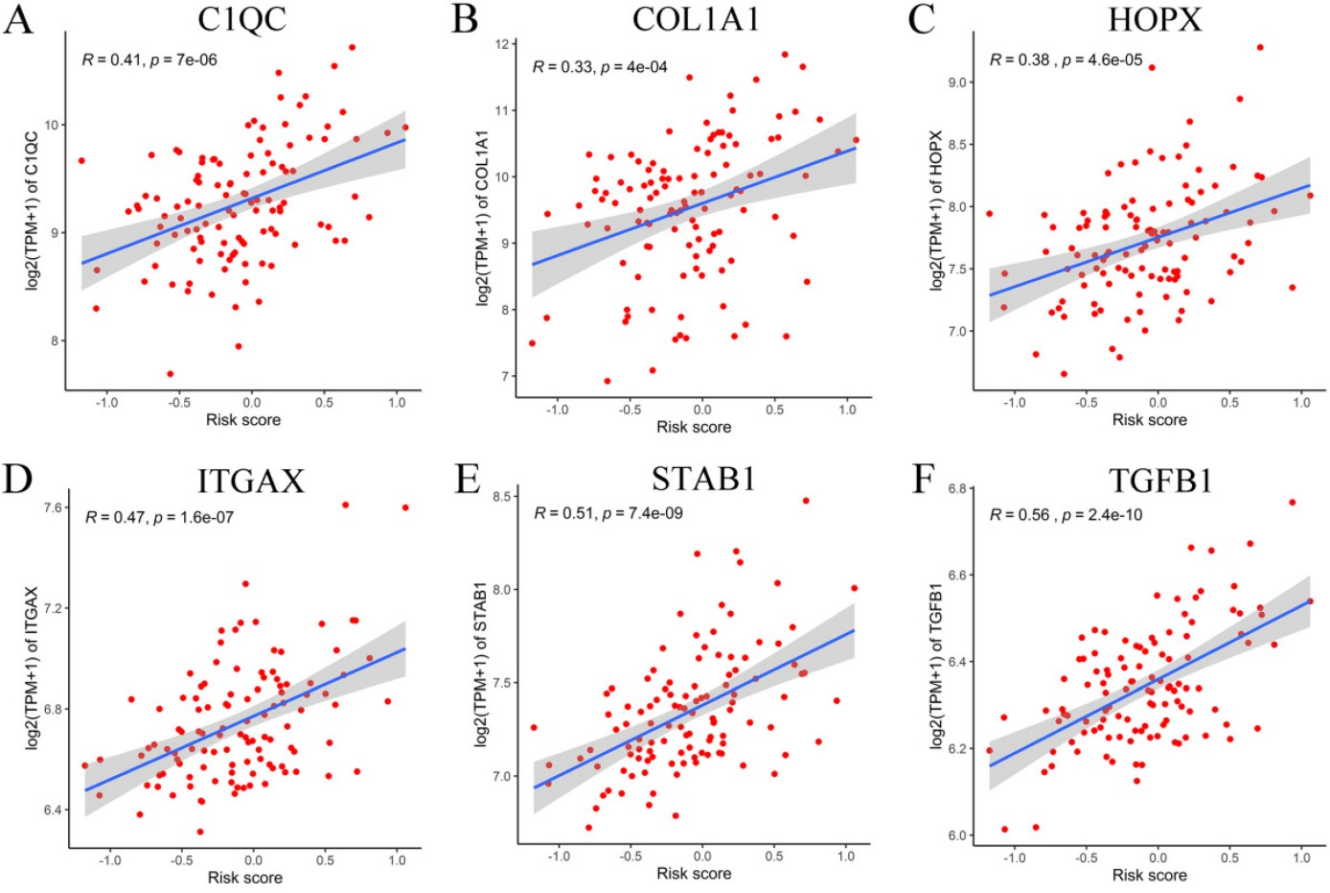
Correlation between six adverse genes and RS in GEO database.** (A-F)** The correlation between six genes and the RS indicated that C1QC, COL1A1, HOPX, ITGAX, STAB1, and TGFB1 were positively correlated with the RS.

**Figure 5:**
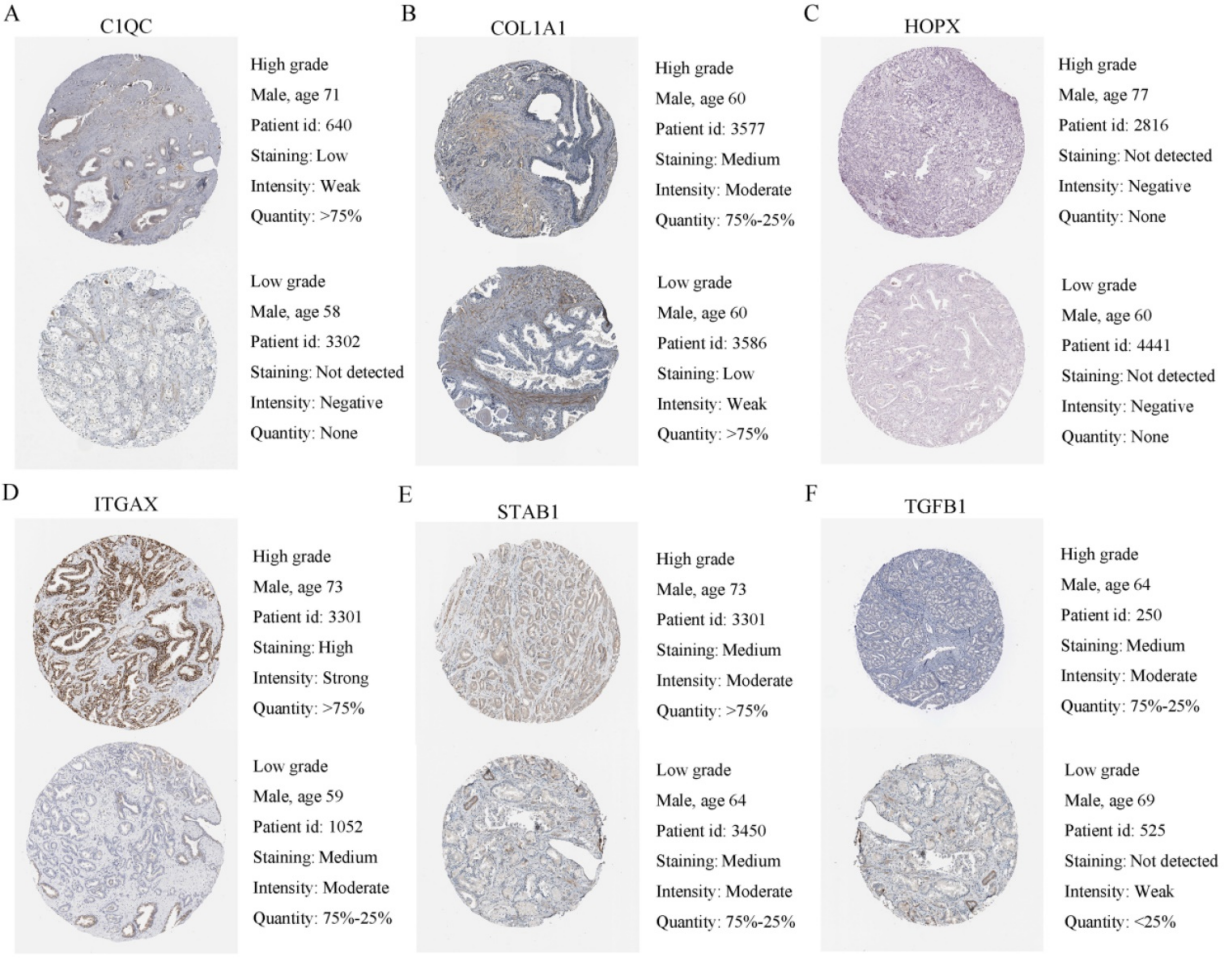
Human protein atlas database was used to explore six adverse expression levels. **(A-F)** Typical IHC of six adverse prognostic genes in high grade and low grade PCa tissues.

To verify the generalization value of the RS model based on the TCGA cohort, we calculated the risk score of each sample for the 111 PCa patients in the GSE70768 cohort using the above mentioned RS formula. The K-M survival curve indicated that the low-RS group had a higher DFS (*P*=0.006) **(Figure 3E)**. In addition, the ROC curves based on the RS model showed that the AUCs for the 1-year, 3-year and 5-year DFS rates were 0.869, 0.859 and 0.835, respectively **(Figure 3F)**. The ROC curves based on the Gleason score model showed that the AUCs for the 1-year, 3-year and 5-year DFS rates were 0.724, 0.695 and 0.688, respectively **(Figure S7D)**. Therefore, the results of the validation set also indicated that the RS model had better robustness and was superior to the Gleason score model. In addition, the correlation between 18 genes and the RS indicated that C1QC, COL1A1, HOPX, ITGAX, STAB1, and TGFB1 were positively correlated with the RS **(Figure 4)**, while the others were negatively correlated** (Figure S8)**. The human protein atlas database was used to explore protein expression levels. Typical IHC of six adverse and eight favorable prognostic genes (except APOF, CHRNA2, FEV, and OLFML3, which are not included in the database) in high grade and low grade PCa tissues are shown in **Figure 5 and Figure S9**, respectively.

### Identification of independent prognostic factors

The univariate Cox model analysis showed that a higher pathological T stage, Gleason score and RS, lymph node metastasis, and positive surgical margin status were risk factors affecting prognosis. In the multivariate Cox analysis, the meaningful variables from the univariate Cox model analysis were included, and the results showed that after the adjustment for variables including variables such as the lymph node status, pathological T stage, Gleason score, and surgical margin status, the RS was independent predictor (HR: 4.04, 95%CI: 0.25-2.44), similar to and independent of the Gleason score** (Table 2)**.

**Figure 6:**
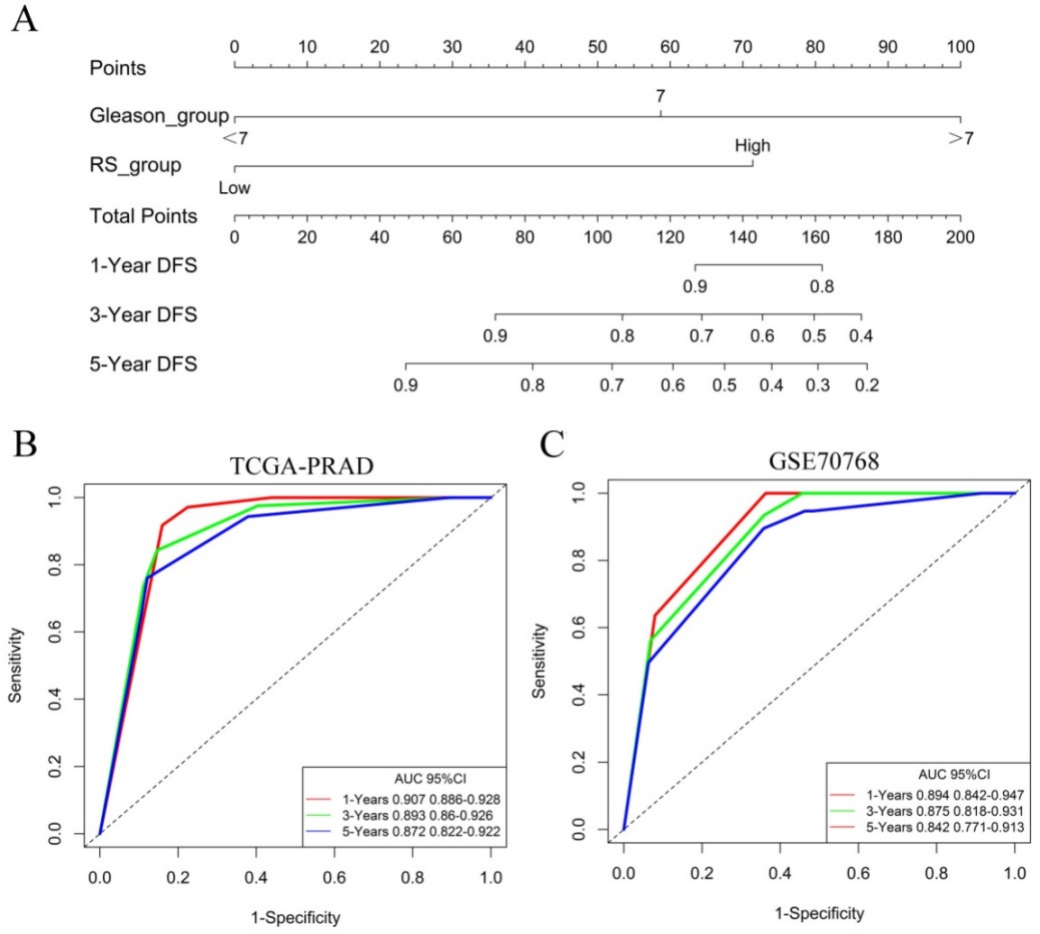
Identification of independent prognostic factors. **(A)** The nomogram for predicting DFS was established based on the RS and Gleason score. **(B)** The AUCs for the 1-year, 3-year and 5-year DFS rates of the nomogram in the TCGA-PRAD dataset.** (C)** The AUCs for the 1-year, 3-year and 5-year DFS rates of the nomogram in the GSE70768 dataset.

The nomogram of the predicted DFS was further established based on the RS and Gleason score **(Figure 6A)**. The AUCs for the 1-year, 3-year and 5-year DFS rates of the nomogram were 0.907 0.893 and 0.872, respectively **(Figure 6B)**. Further validation of an independent cohort of 111 PCa patients in the GSE70768 cohort also showed good predictive power** (Figure 6C)**.

### Estimating the composition of immune cells

**Figure 7:**
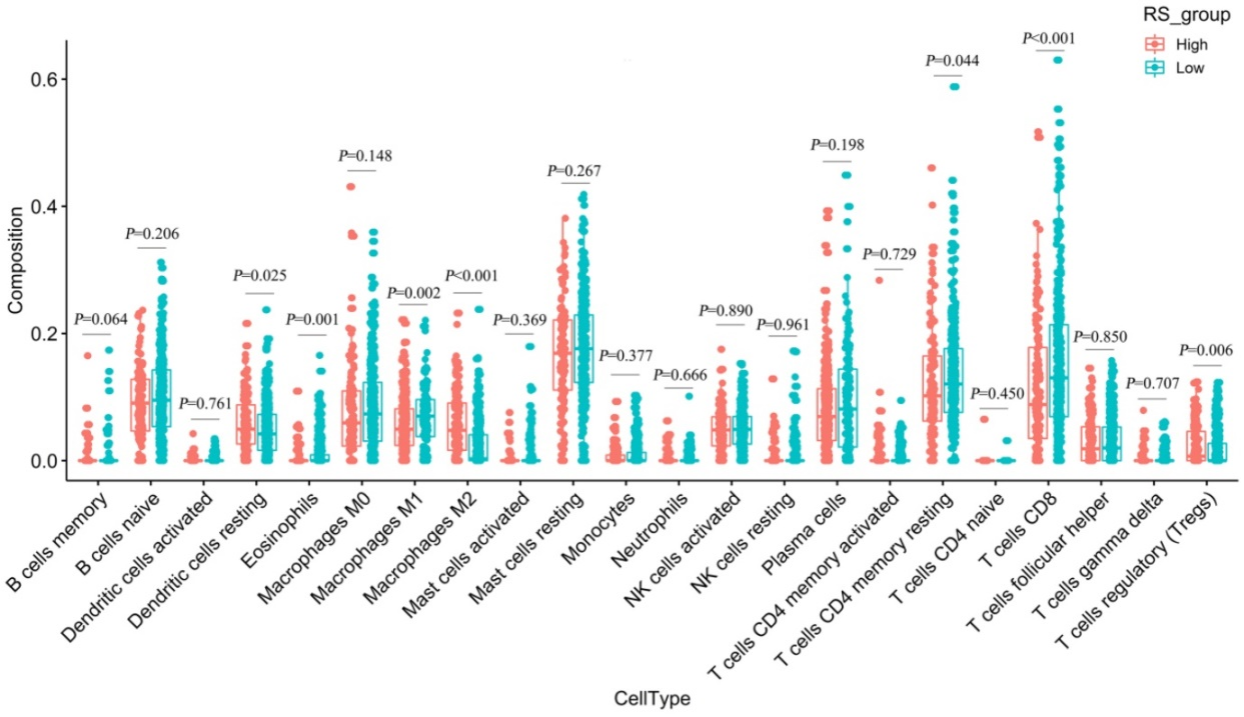
Differences in 22 immune cells between the high-RS and low-RS groups.

We used CIBERSORT to estimate the immune cell composition of 480 samples and to quantify the relative levels of different cell types in the mixed cell population **(Figure S10)**. As shown in **Figure 7**, we compared different cell types of patients in the low-RS group with those in the high-RS group. These results indicated that the expression levels of resting memory CD 4 T cells, CD 8 T cells, M1 macrophages and eosinophils in the low-risk group were significantly higher than those in the high-risk group (*P*<0.05). In contrast, the expression levels of M2 macrophages, regulatory T cells (Tregs) and dendritic cells resting in the high-RS group were significantly higher than those in the low-RS group (*P*<0.05).

**Figure 8:**
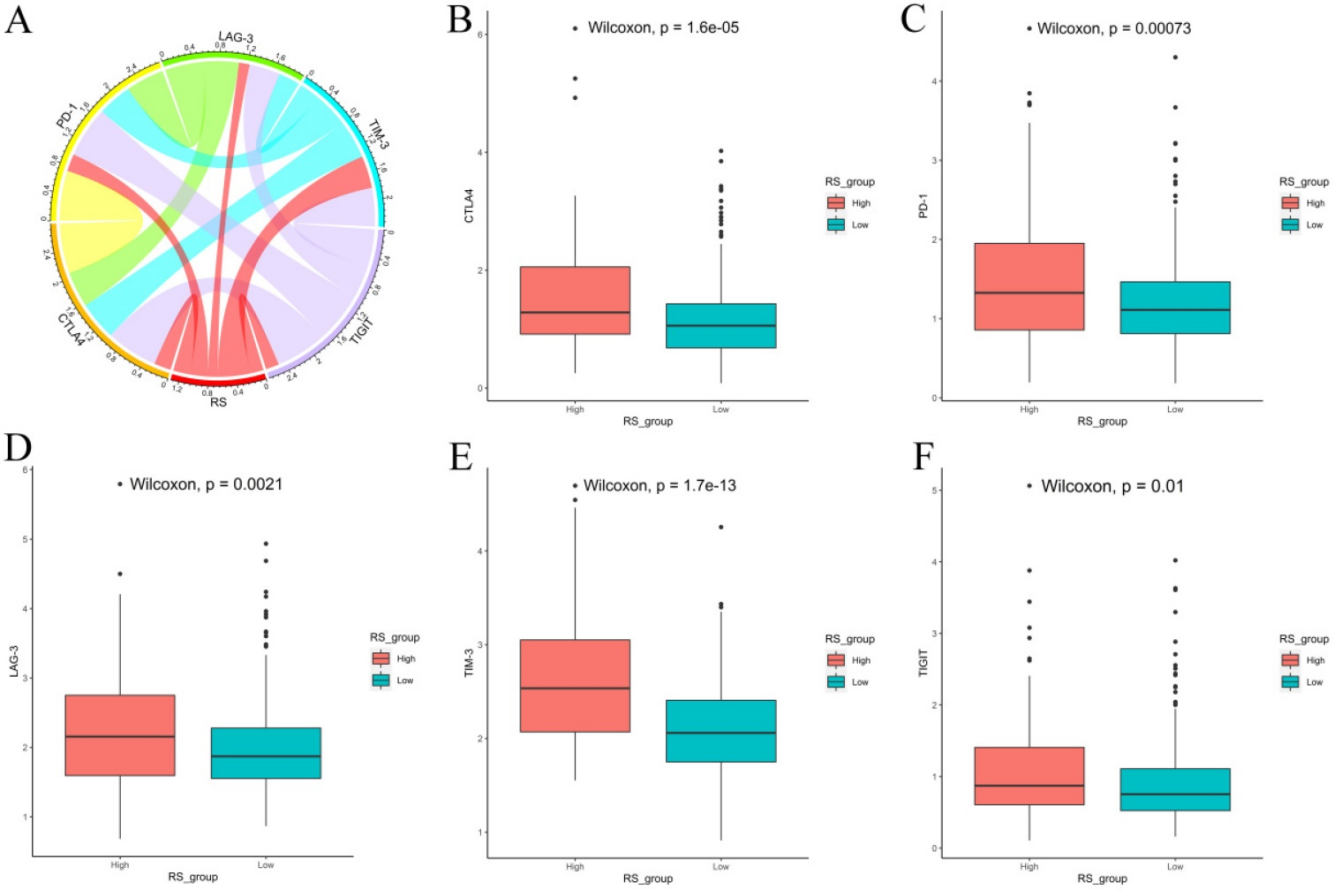
Expression of immune checkpoints between high-RS and low-RS PCa patients. **(A)** Correlation of the RS with the expression levels of several prominent immune checkpoints. **(B-F)** Box figure showing significantly different immune checkpoints between high-RS and low-RS patients.

We also explored the expression of immune checkpoints between the high-RS and low-RS PCa patients. The expression levels of CTLA-4, PD-1, LAG-3, TIM-3 and TIGIT in high-RS patients were significantly higher than those in low-RS patients (*P*<0.05) **(Figure 8)**.

## Discussion

Previous studies have shown that the TME plays a vital role in the development, progression and recurrence of cancer 20, 21. However, due to the heterogeneity and complexity of the TME, only some PCa patients benefit from immunotherapy. It remains critical to construct an effective model for accurately predicting the prognosis of PCa patients. To the best of our knowledge, this study is the first to apply the ESTIMATE algorithm to identify a TME-related prognostic signature of PCa patients after RP.

In our study, we calculated the immune/stromal score of each PCa sample extracted from the TCGA database by applying an ESTIMATE algorithm. The results showed that a higher immune/stromal score was associated with a poorer DFS, higher Gleason score, and higher pathological T stage in PCa patients. Subsequently, we divided PCa patients into high/low immune (or stromal) score groups and identified 515 cross-sectional DEGs. The GO and KEGG analysis of DEGs showed that DEGs primarily participated in the TME, such as immune responses, inflammatory responses, cell adhesion, extracellular matrix organization, and leukocyte migration. These processes may inhibit tumor progression and metastasis, thereby improving DFS. We found that these DEGs have a strong correlation with the immune response and tumor immune microenvironment. In addition, we applied univariate Cox and LASSO Cox regression models to construct a prognostic signature model based on 18 DEGs. In this model, the DFS in the high-RS group was significantly lower than that in the low-risk group, and the AUCs for the 1-year, 3-year and 5-year DFS rates were 0.890, 0.877 and 0.841, respectively; thus, recurrence in PCa patients could be well predicted. In addition, the stratified analysis showed that the RS model also had strong prognostic capability for PCa patients with negative surgical margins (R0). Our model has more advantages in predicting the accuracy of prognosis than other models in other studies (the AUC of the ROC curve varied from 0.605-0.768) 22-24 and is expected to be applied to the clinical prognosis assessment of PCa patients.

Among the RS models, the expression levels of C1QC, COL1A1, HOPX, ITGAX, STAB1, and TGFB1 were low, and the prognosis was good. In contrast, we found that the low expression levels of other genes were related to the poor prognosis of PCa patients. C1QC belongs to C1Q and plays an important role in adaptive and innate immune responses. Studies have shown that C1QC can promote the adhesion, migration and proliferation of malignant pleural mesothelioma 25, as well as the increase in C1QC levels in patients with sarcoma, and C1Q1 is associated with poor prognosis 26. High expression of the COL1A1 gene will cause unrestricted growth factors which, in turn, will benefit tumor proliferation 27. The HOPX gene may be involved in the malignant transformation of cancer cells. Studies have shown that higher HOPX expression is an independent adverse prognostic factor for acute myeloid leukemia 28. The expression level of ITGAX is positively correlated with aggressive prostate cancer 29. STAB1 is an identified oncogene whose increased expression promotes tumorigenesis and tumor progression 30, and it is associated with poor prognosis in many cancers. TGFB1 is often up-regulated in tumor cells and highly secreted into the prostate environment, partially mediating the immunosuppressive effect on NK cells and promoting the invasion and metastasis of prostate cancer 31.

APOF can act as a tumor suppressor for hepatocellular carcinoma, and the decreased expression of APOF is associated with a poor prognosis 32. The genetic variation in the nicotinic cholinergic receptor gene (CHRNS) may affect the risk of lung cancer 33. The low expression of CLIC6 in breast cancer is related to a high histological grade 34. The tumor suppressor gene EGR-1 can directly mediate the apoptotic function through the transcriptional upregulation of Bax-mRNA and protein and the increase of oligomerization and activation 35. FEV is rich in alanine c-terminal, indicating that it may act as a transcriptional inhibitor 36. FOS is considered a regulator of cell proliferation, differentiation, and transformation and participates in the MAPK signaling pathway 37, 38. GJB1 is abundantly expressed in other well-differentiated cell types such as prostate and pancreas. In prostate tumors, the ability to assemble GJS from GJB1 and GJA1 is impaired 39. GNG2 is involved in the signal transduction of the GPCR and CCR5 pathways in macrophages, and the expression level of GNG2 in malignant melanoma is decreased 40, 41. The protein encoded by the HSD11b1 gene is a microsomal enzyme involved in the synthesis and regulation of prostaglandins. Up-regulation of OLFML3 enhances self-renewal of glioma stem cells and triggers primary tumor immunity, and PLTP plays a crucial role in mediating the association between triacyl lipid A and lipoprotein, which is beneficial to the anticancer properties 42. As a candidate cancer suppressor, low TGM3 expression is associated with a poor overall survival rate in patients with neck cancer 43.

The RS prognostic model constructed by these 18 genes has not been reported and may represent a new prognostic factor for PCa. Furthermore, the multivariate Cox model showed that the RS and Gleason score were two independent prognostic indicators. To provide personalized scores for the prognosis of each PCa patient, a nomogram combining the TME-related RS and Gleason score for the prediction of DFS rate was established. The AUCs for the 1-year, 3-year and 5-year DFS rates of the nomogram were 0.907 0.893 and 0.872, respectively. However, the ROC curves based on the Gleason score model only showed that the AUCs for the 1-year, 3-year and 5-year DFS rates were 0.724, 0.695 and 0.688, respectively. The combination of the TME-related RS and Gleason score was shown to have better prognostic value than the Gleason score alone.

Finally, we used CIBERSORT and LM22 to jointly estimate the scores of 22 human immune cell types in PCa samples, and compared differences in the composition of immune cell types and the expression levels of five immune checkpoints (CTLA-4, PD-1, LAG-3, TIM-3 and TIGIT) between high-RS and low-RS groups. The concentrations of M2 macrophages and Tregs were higher in the high-RS group. In contrast, the low-RS group had a higher proportion of CD 8 T cells, resting memory CD 4 T cells, and M1 macrophages. The expressions levels of CTLA-4, PD-1, LAG-3, TIM-3 and TIGIT in high-RS patients were significantly higher than those in low-RS patients (*P*<0.05). Previous studies have shown that resting memory CD 4 T cells can further differentiate and have multiple functions, including restoring immune tolerance to autoantigens or heteroantigens and promoting CD 8 T cells actions against tumors 44, 45. Tregs expressing CTLA-4 play a crucial role in the maintenance of immunological self-tolerance and homeostasis and suppressing the anti-tumor immune response 46. CTLA-4 is expressed in activated CD 4 and CD 8 T cells, which can terminate the response of activated T cells and mediate the inhibitory function of Tregs 47. Overexpression of PD-1 on CD 8 T cells is an indicator of T-cell depletion 48. Inhibiting or knocking out LAG-3 will release the inhibitory function of Tregs on T cells. TIM-3 suppresses anti-tumor immunity by mediating T-cell depletion. TIGIT can suppress immune cells in multiple steps of the tumor immune cycle 49. In our study, the proportion of Tregs in high-RS patients was higher, the expression levels of the immune checkpoints CTLA-4, PD-1, LAG-3, TIM-3 and TIGIT were higher, and the prognosis was poor, suggesting that the immunosuppressive environment and the high expression of immune checkpoints may be the reasons for the poor prognosis of PCa. In addition, these results suggested that anti-CTLA4 drugs blocking immune checkpoints leads to T-cell activation, which is an ideal strategy for treating cancer. Anti-immune checkpoint antibody treatment will be more beneficial to high-risk PCa patients than low-risk patients, resulting in a better prognosis.

However, this study also has certain limitations. First, this study only conducted bioinformatics research using public databases. Next, we should verify the results of this study using clinical patients in a study with the prospective design. Second, the 18 hub genes related to immune cell infiltration should be further studied to clarify the regulatory mechanism of PCa immune infiltration.

## Conclusion

Our study established and validated a model of RS based on 18 TME-related genes, which provided a theoretical basis for predicting the DFS of PCa and further demonstrated the TME-related features associated with tumor immune cell infiltration. These genes may be of great significance for the individualization of treatment and immunotherapy for PCa patients and postoperative rehabilitation management.

## Supplementary Material

Supplementary figures.

## Figures and Tables

**Table 1:** Clinical characteristics of 480 PCa patients included in the study from the TCGA cohort

Variables	Whole cohort (N=480)	Relapse or death		Log-rank *P*
		No (N=386)	Yes (N=94)	
**Age**				0.462
<65	319 (66.46)	257 (80.56)	62 (19.44)	
≥65	161 (33.54)	129 (80.12)	32 (19.88)	
**Tumor laterality**				0.212
Left/Right	56 (11.67)	48 (85.71)	8 (14.29)	
Bilateral	424 (88.33)	338 (79.72)	86 (20.28)	
**Pathological T stage**				<0.001
≤pT2c	186 (38.75)	170 (91.40)	16 (8.60)	
pT3a	153 (31.88)	120 (78.43)	33 (21.57)	
≥pT3b	141 (29.38)	96 (68.09)	45 (31.91)	
**Lymph node status**				0.007
pN0/pNx	402 (83.75)	331 (82.34)	71 (17.66)	
pN1	78 (16.25)	55 (70.51)	23 (29.49)	
**Gleason score**				<0.001
<7	44 (9.17)	43 (97.73)	1 (2.27)	
7	240 (50.00)	214 (89.17)	26 (10.83)	
>7	196 (40.83)	129 (65.82)	67 (34.18)	
**Surgical margin status**				<0.001
Negative	333 (69.38)	283 (84.98)	50 (15.02)	
Positive	147 (30.63)	103 (70.07)	44 (29.93)	

**Table 2:** Univariate and multivariate Cox analysis of clinical information and the RS

Variables	Univariate analysis		Multivariate analysis	
	HR (95%CI)	*P*	HR (95%CI)	*P*
**Age**				
<65	1			
≥65	0.46 (0.77-1.8)	0.462		
**Tumor laterality**				
Left/Right	1			
Bilateral	1.58 (0.77-3.27)	0.215		
**Pathological T stage**				
≤pT2c	1		1	
pT3a	1.8 (1.43-4.77)	0.002	1.49 (0.79-2.8)	0.222
≥pT3b	4.59 (2.57-8.2)	<0.001	1.16 (0.56-2.37)	0.691
**Lymph node status**				
pN0/pNx	1		1	
pN1	1.89 (1.18-3.03)	0.009	0.65 (1.55-0.39)	0.098
**Gleason score**				
<7	1		1	
7	4.73 (0.64-34.89)	0.127	3.88 (0.26-0.52)	0.185
>7	17.34 (2.41-125)	0.005	8.5 (0.12-1.13)	0.038
**Surgical margin status**				
Negative	1		1	
Positive	2.34 (1.56-3.52)	<0.001	1.51 (0.66-0.97)	0.066
**Risk score**				
Low	1		1	
High	5.9 (3.88-8.97)	<0.001	4.04 (0.25-2.44)	<0.001

## Abbreviations

TME: tumor microenvironment
PCa: prostate cancer
RP: radical prostatectomy
TCGA: The Cancer Genome Atlas
GEO: Gene Expression Omnibus
ESTIMATE: Estimation of Stromal and Immune cells in Malignant Tumor tissues using Expression data
CIBERSORT: Cell type Identification by Estimating Relative Subpopulations of RNA Transcription
FPKM: fragments per kilobase million
TPM: transcripts per million
GDC: Genomic Data Commons
DEGs: differentially expressed genes
GO: Gene ontology
BPs: biological processes
MFs: molecular functions
CCs: cellular components
KEGG: Kyoto Encyclopedia of Genes and Genomes
IHC: immunohistochemistry
DFS: disease-free survival
LASSO: least absolute shrinkage and selection operation
RS: risk score
Coeff: coefficient
ROC: receiver operating characteristic
AUC: under the curve
K-M: Kaplan-Meier
FDR: false discovery rate
HR: hazard ratio
