# Supplementary material for: Identification of a Prognostic Signature Model with Tumor Microenvironment for predicting Disease-free Survival after Radical Prostatectomy
Source: J Cancer. 2021 Feb 22;12(8):2371–84. doi: 10.7150/jca.51173 (PMC7974886; doi:10.7150/jca.51173)
Supplement: Supplementary file 1 — Supplementary figures. [file jcav12p2371s1.pdf]

Figure S1. The workflow chart of study design.

Figure S2. Relationship between the estimate/stromal score and clinical characteristics. (**A-D**) The relationship between the estimate score and T stage, surgical marginal status, Gleason score, and tumor laterality. **(E)** The relationship between stromal score and tumor laterality.

Figure S3. Volcano plot used to screen for DEGs. **(A)** The volcano plot shows DEGs between high and low immune scores. (**B)** The volcano plot shows DEGs between high and low stromal scores.

Figure S4. The name and coefficient of the 18-gene signature closely related to TME.

Figure S5. Survival curve of high and low expression of six adverse prognostic genes. **(A-F)** Patients with low expression levels of C1QC, COL1A1, HOPX, ITGAX, STAB1, and TGFB1 had a better prognosis.

Figure S6. Survival curve of high and low expression of twelve favorable prognostic genes. **(A-L)** Patients with high expression levels of APOF, CLIC6, COL1A1, EGR1, FEV, FOS, GJB1, GNG2, HSD11B1, OLFML3, PLTP, and TGFB1 had a better prognosis.

Figure S7. (A) For sensitivity verification, a K-M curve based on the median RS grouping in the TCGA-PRAD dataset was used. **(B)** In the absence of residual tumors, a K-M curve for the low-RS and high-RS groups in the TCGA-PRAD dataset was used. **(C)** ROC curve based on the Gleason score model in the TCGA-PRAD dataset. **(D)** ROC curve based on the Gleason score model in the GSE70768 dataset.

Figure S8. Correlation between twelve favorable genes and the RS in the GSE70768 dataset. **(A-F)** The correlation between twelve genes and the RS indicated that C1QC, COL1A1, HOPX, ITGAX, STAB1, and TGFB1 were positively correlated with the RS.

Figure S9. Human protein atlas database was used to explore eight favorable expression levels. **(A-F)** Typical IHC of eight favourable prognostic genes in high- and low- grade PCa tissues.

Figure S10. Estimating the composition of immune cells. **(A)** Relative proportion of 22 immune cells infiltrating in high-RS and low-RS patients. **(B)** Heat map of the 22 immune cells proportions in high-RS and low-RS patients.

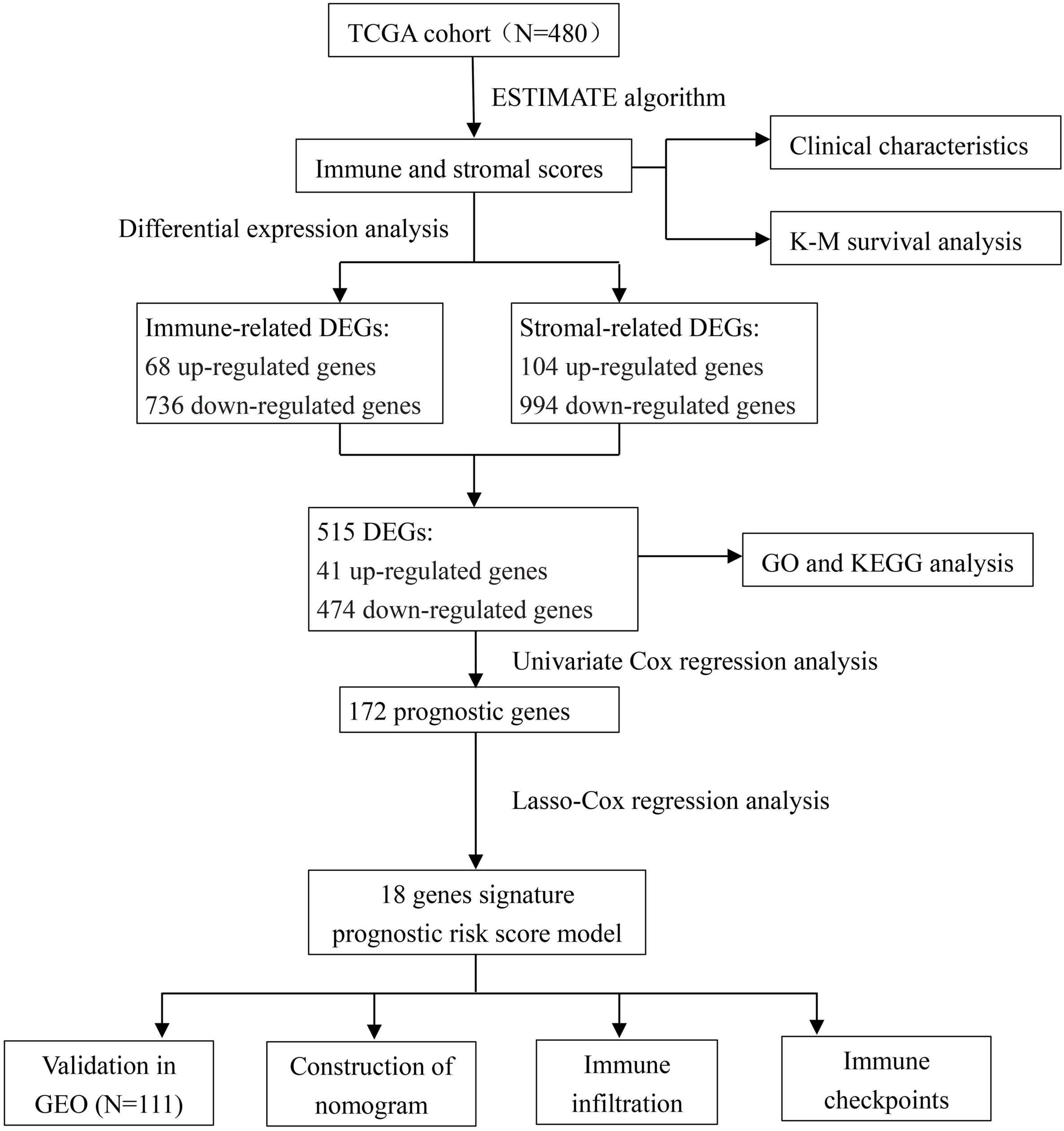
TCGA cohort（N=480）
ESTIMATE algorithm
Immune and stromal scores
Clinical characteristics
K-M survival analysis
Differential expression analysis
Immune-related DEGs:
68 up-regulated genes
736 down-regulated genes
Stromal-related DEGs:
104 up-regulated genes
994 down-regulated genes
515 DEGs:
41 up-regulated genes
474 down-regulated genes
GO and KEGG analysis
Univariate Cox regression analysis
172 prognostic genes
Lasso-Cox regression analysis
18 genes signature
prognostic risk score model
Validation in
GEO (N=111)
Construction of
nomogram
Immune
infiltration
Immune
checkpoints

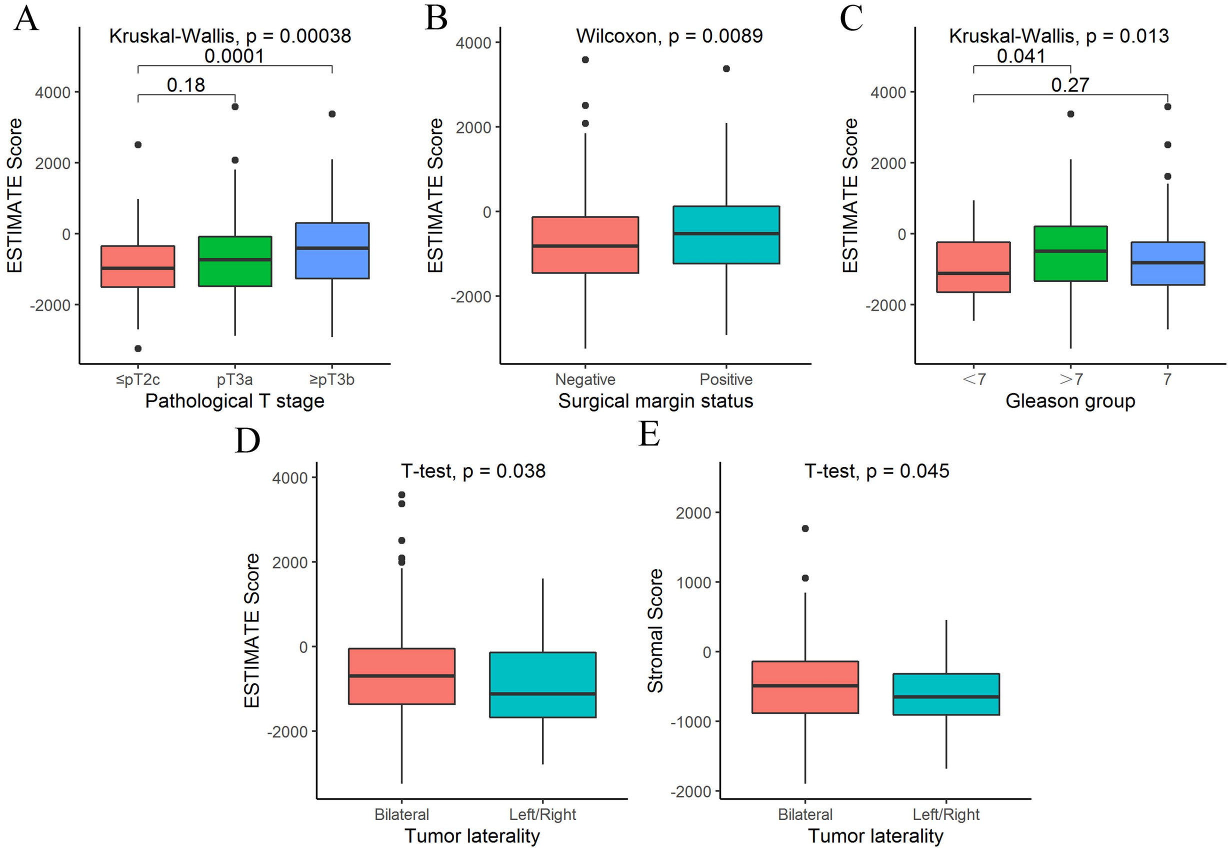
A
Kruskal-Wallis, p = 0.00038
0.0001
0.18
ESTIMATE Score
4000
2000
0
-2000
≤pT2c
pT3a
≥pT3b
Pathological T stage
B
Wilcoxon, p = 0.0089
ESTIMATE Score
4000
2000
0
-2000
Negative
Positive
Surgical margin status
C
Kruskal-Wallis, p = 0.013
0.041
0.27
ESTIMATE Score
4000
2000
0
-2000
<7
>7
7
Gleason group
D
T-test, p = 0.038
ESTIMATE Score
4000
2000
0
-2000
Bilateral
Left/Right
Tumor laterality
E
T-test, p = 0.045
Stromal Score
2000
1000
0
-1000
-2000
Bilateral
Left/Right
Tumor laterality

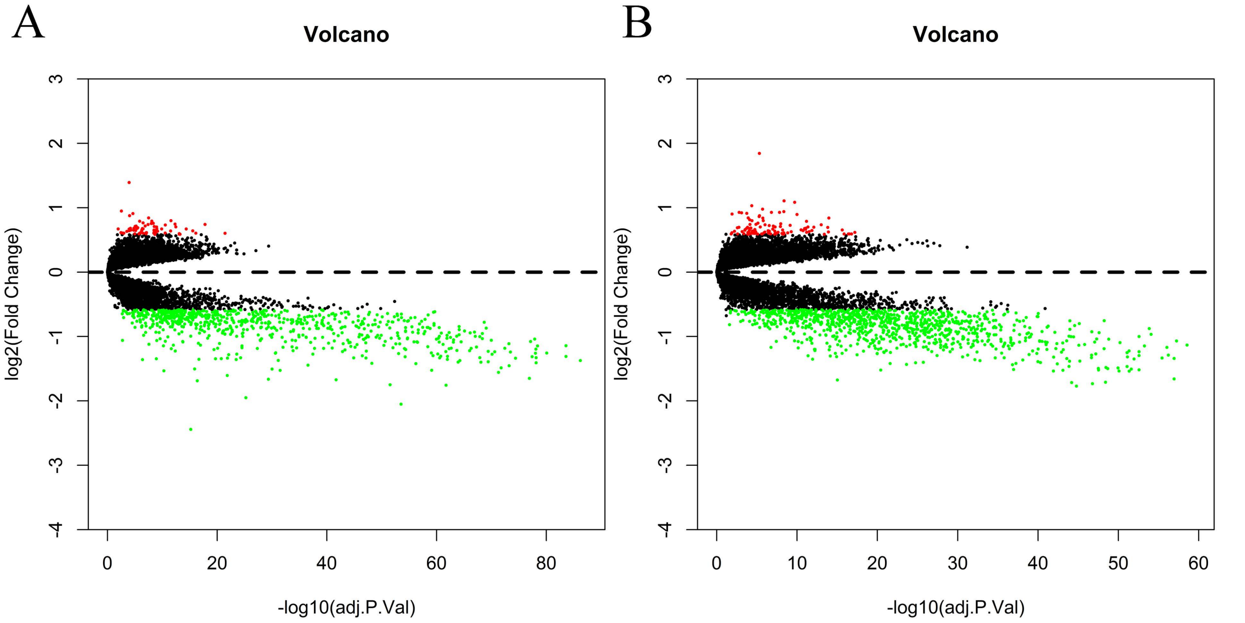

A
Volcano
log2(Fold Change)
3
2
1
0
-1
-2
-3
-4
0
20
40
60
80
-log10(adj.P.Val)
B
Volcano
log2(Fold Change)
3
2
1
0
-1
-2
-3
-4
0
10
20
30
40
50
60
-log10(adj.P.Val)

Gene	Coeff	Gene	Coeff
C1QC	0.06410	EGR1	-0.03191
COL1A1	0.00467	FEV	-0.01361
HOPX	0.01777	FOS	-0.00269
ITGAX	0.17512	GJB1	-0.06470
STAB1	0.35768	GNG2	-0.23936
TGFB1	0.13676	HSD11B1	-0.10059
APOF	-0.04815	OLFML3	-0.03021
CHRNA2	-0.06180	PLTP	-0.06134
CLIC6	-0.22487	TGM3	-0.03940

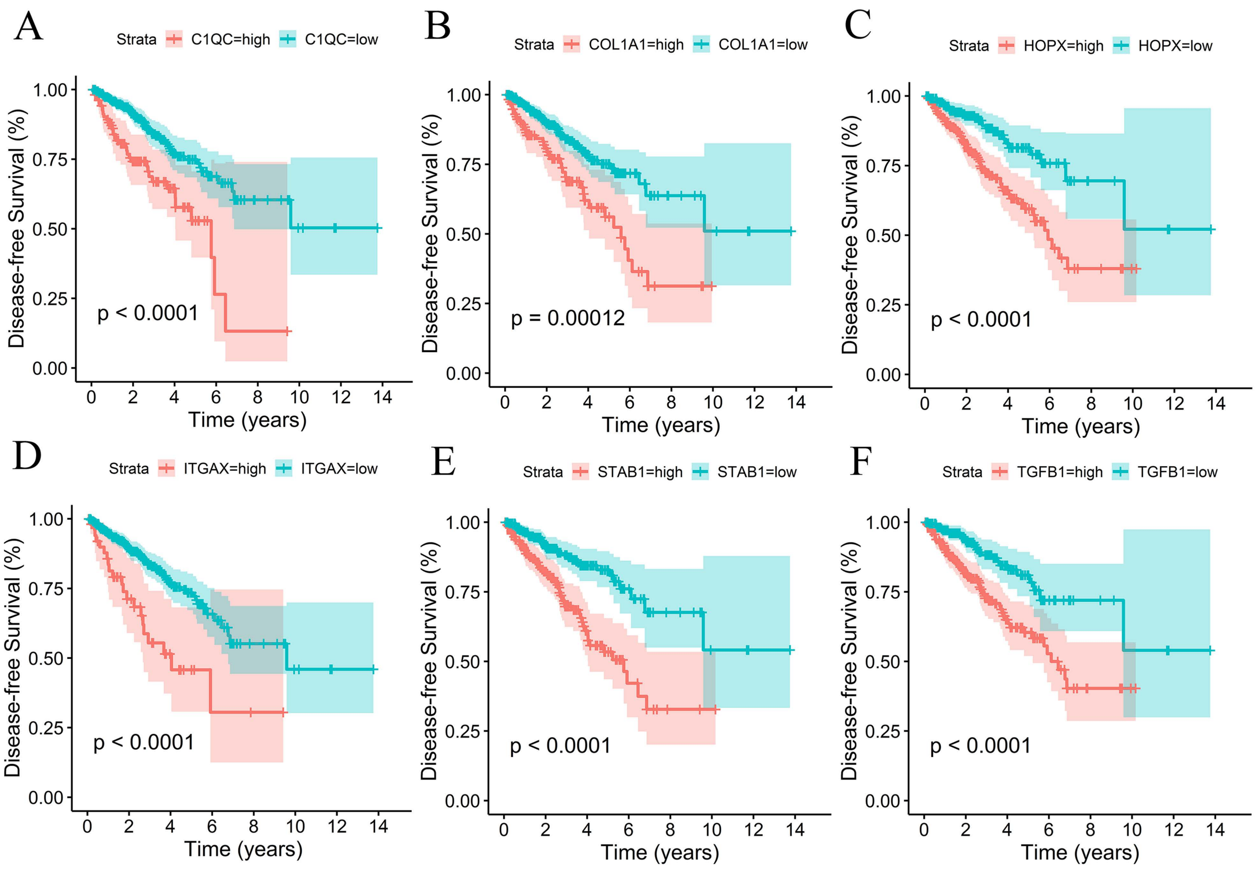
A
Strata C1QC=high C1QC=low
p < 0.0001
B
Strata COL1A1=high COL1A1=low
p = 0.00012
C
Strata HOPX=high HOPX=low
p < 0.0001
D
Strata ITGAX=high ITGAX=low
p < 0.0001
E
Strata STAB1=high STAB1=low
p < 0.0001
F
Strata TGFB1=high TGFB1=low
p < 0.0001
Disease-free Survival (%)
Time (years)
1.00
0.75
0.50
0.25
0.00
0
2
4
6
8
10
12
14

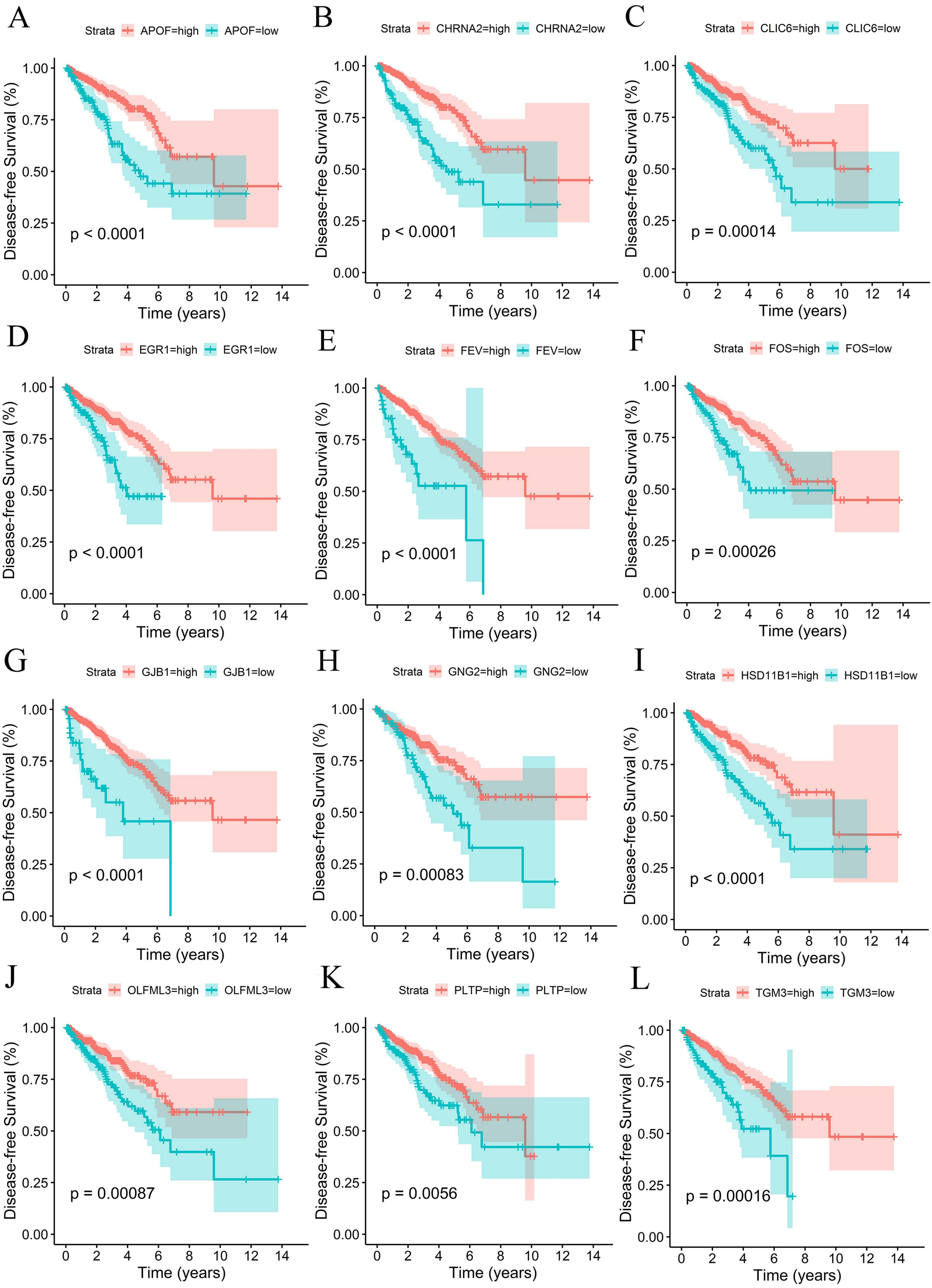
A
Strata APOF=high APOF=low
Disease-free Survival (%)
Time (years)
p < 0.0001
B
Strata CHRNA2=high CHRNA2=low
p < 0.0001
C
Strata CLIC6=high CLIC6=low
p = 0.00014
D
Strata EGR1=high EGR1=low
p < 0.0001
E
Strata FEV=high FEV=low
p < 0.0001
F
Strata FOS=high FOS=low
p = 0.00026
G
Strata GJB1=high GJB1=low
p < 0.0001
H
Strata GNG2=high GNG2=low
p = 0.00083
I
Strata HSD11B1=high HSD11B1=low
p < 0.0001
J
Strata OLFML3=high OLFML3=low
p = 0.00087
K
Strata PLTP=high PLTP=low
p = 0.0056
L
Strata TGM3=high TGM3=low
p = 0.00016

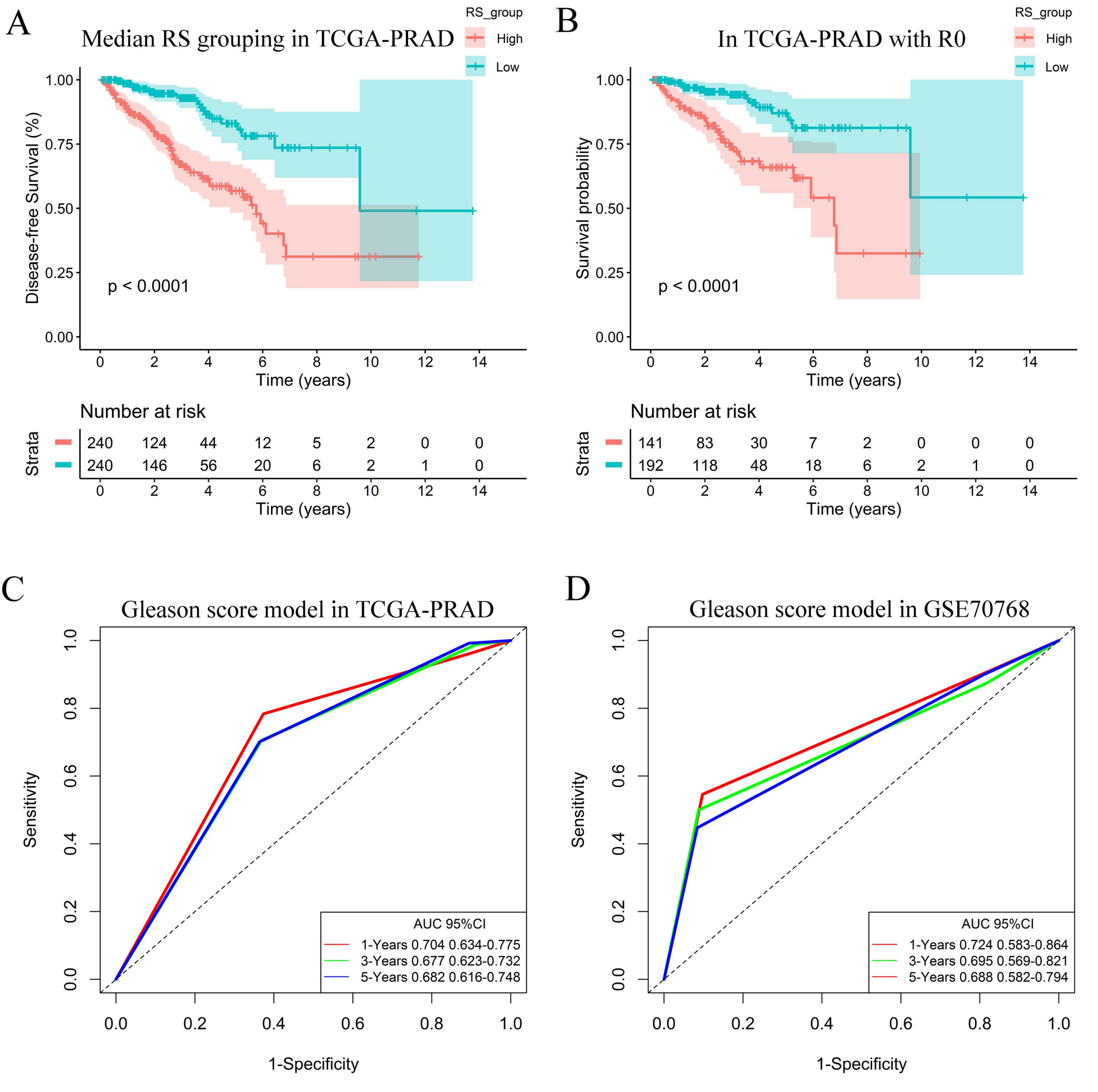

A
Median RS grouping in TCGA-PRAD
RS_group
High
Low
Disease-free Survival (%)
1.00
0.75
0.50
0.25
0.00
p < 0.0001
Time (years)
0 2 4 6 8 10 12 14
Number at risk
Strata
240 124 44 12 5 2 0 0
240 146 56 20 6 2 1 0
B
In TCGA-PRAD with R0
Survival probability
p < 0.0001
Number at risk
141 83 30 7 2 0 0 0
192 118 48 18 6 2 1 0
C
Gleason score model in TCGA-PRAD
Sensitivity
1-Specificity
AUC 95%CI
1-Years 0.704 0.634-0.775
3-Years 0.677 0.623-0.732
5-Years 0.682 0.616-0.748
D
Gleason score model in GSE70768
AUC 95%CI
1-Years 0.724 0.583-0.864
3-Years 0.695 0.569-0.821
5-Years 0.688 0.582-0.794

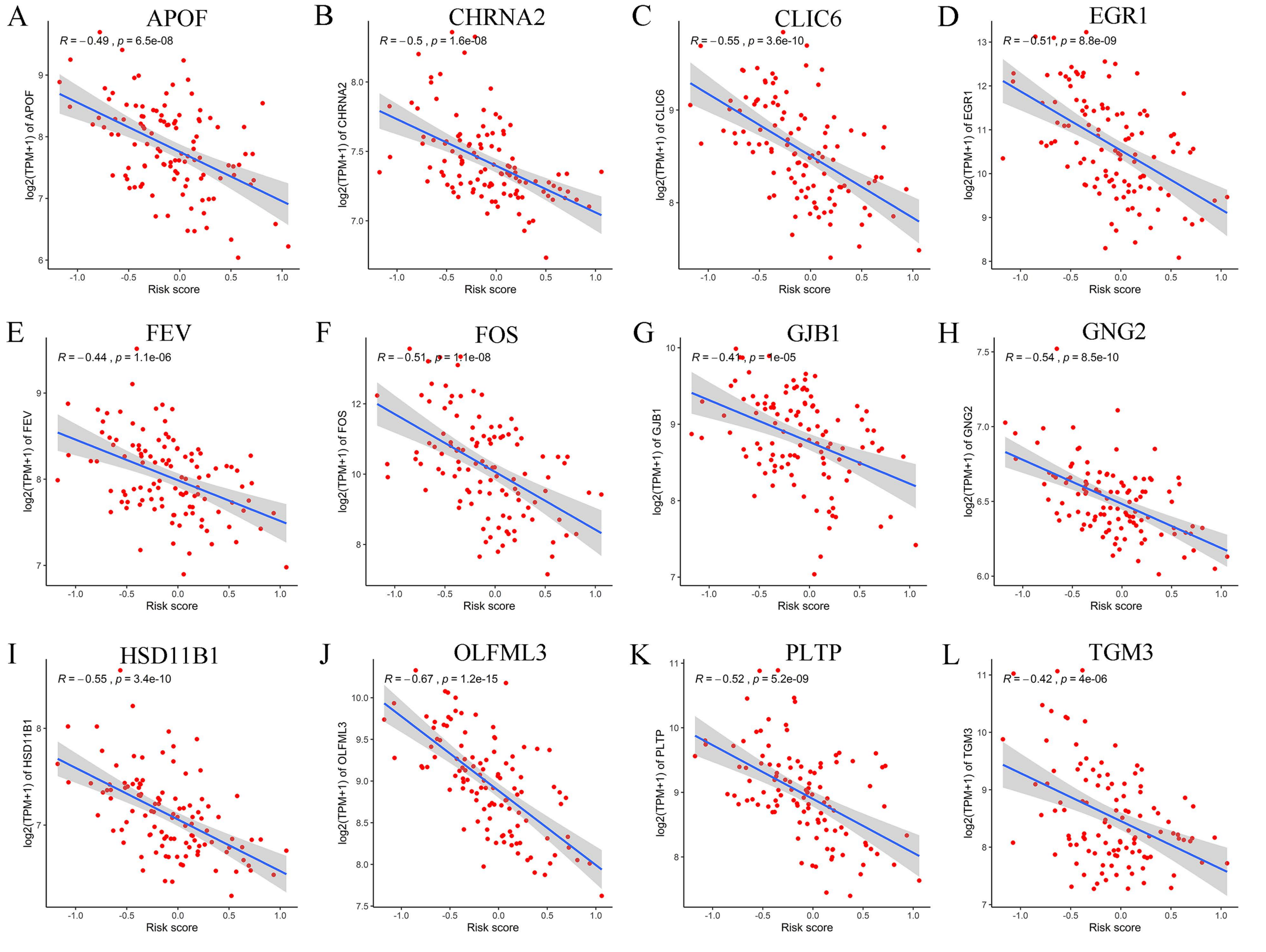

A
APOF
R = −0.49 , p = 6.5e-08
log2(TPM+1) of APOF
Risk score
B
CHRNA2
R = −0.5 , p = 1.6e-08
log2(TPM+1) of CHRNA2
Risk score
C
CLIC6
R = −0.55 , p = 3.6e-10
log2(TPM+1) of CLIC6
Risk score
D
EGR1
R = −0.51 , p = 8.8e-09
log2(TPM+1) of EGR1
Risk score
E
FEV
R = −0.44 , p = 1.1e-06
log2(TPM+1) of FEV
Risk score
F
FOS
R = −0.51 , p = 1.1e-08
log2(TPM+1) of FOS
Risk score
G
GJB1
R = −0.41 , p = 1e-05
log2(TPM+1) of GJB1
Risk score
H
GNG2
R = −0.54 , p = 8.5e-10
log2(TPM+1) of GNG2
Risk score
I
HSD11B1
R = −0.55 , p = 3.4e-10
log2(TPM+1) of HSD11B1
Risk score
J
OLFML3
R = −0.67 , p = 1.2e-15
log2(TPM+1) of OLFML3
Risk score
K
PLTP
R = −0.52 , p = 5.2e-09
log2(TPM+1) of PLTP
Risk score
L
TGM3
R = −0.42 , p = 4e-06
log2(TPM+1) of TGM3
Risk score

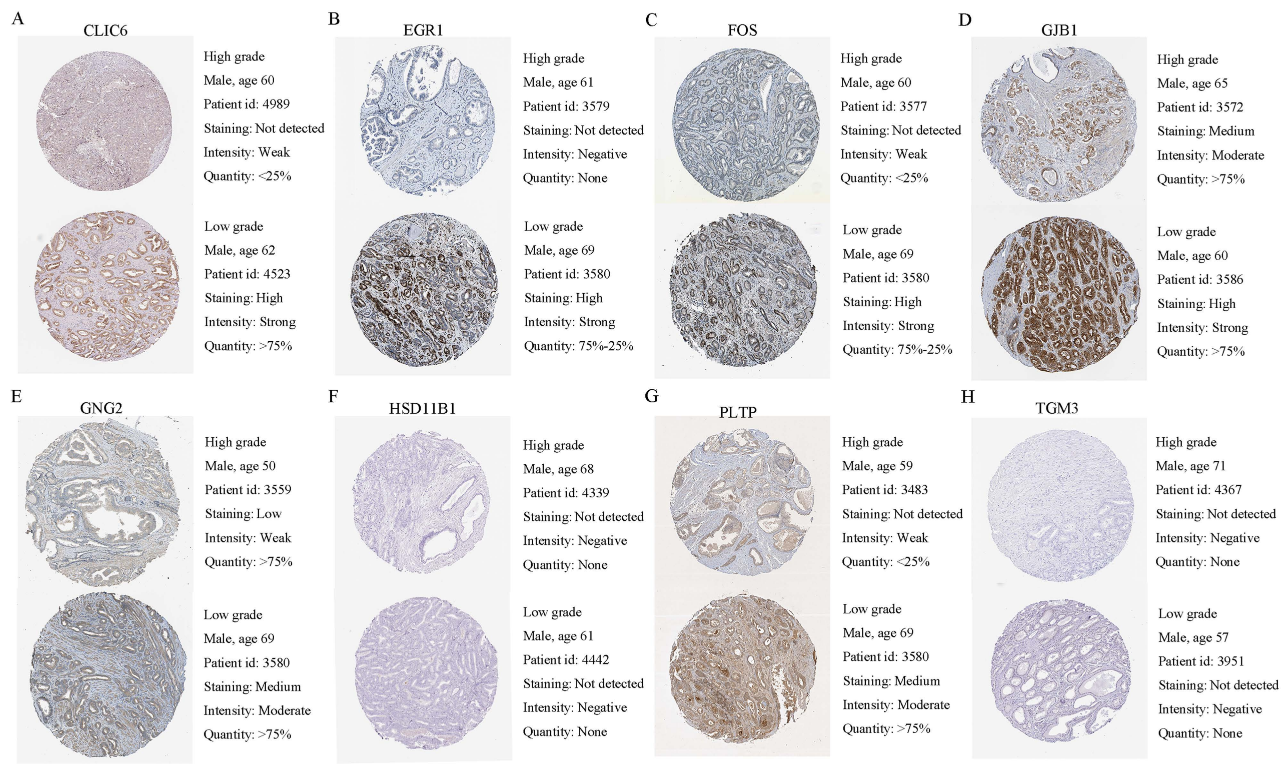
A
CLIC6
High grade
Male, age 60
Patient id: 4989
Staining: Not detected
Intensity: Weak
Quantity: <25%
Low grade
Male, age 62
Patient id: 4523
Staining: High
Intensity: Strong
Quantity: >75%
B
EGR1
High grade
Male, age 61
Patient id: 3579
Staining: Not detected
Intensity: Negative
Quantity: None
Low grade
Male, age 69
Patient id: 3580
Staining: High
Intensity: Strong
Quantity: 75%-25%
C
FOS
High grade
Male, age 60
Patient id: 3577
Staining: Not detected
Intensity: Weak
Quantity: <25%
Low grade
Male, age 69
Patient id: 3580
Staining: High
Intensity: Strong
Quantity: 75%-25%
D
GJB1
High grade
Male, age 65
Patient id: 3572
Staining: Medium
Intensity: Moderate
Quantity: >75%
Low grade
Male, age 60
Patient id: 3586
Staining: High
Intensity: Strong
Quantity: >75%
E
GNG2
High grade
Male, age 50
Patient id: 3559
Staining: Low
Intensity: Weak
Quantity: >75%
Low grade
Male, age 69
Patient id: 3580
Staining: Medium
Intensity: Moderate
Quantity: >75%
F
HSD11B1
High grade
Male, age 68
Patient id: 4339
Staining: Not detected
Intensity: Negative
Quantity: None
Low grade
Male, age 61
Patient id: 4442
Staining: Not detected
Intensity: Negative
Quantity: None
G
PLTP
High grade
Male, age 59
Patient id: 3483
Staining: Not detected
Intensity: Weak
Quantity: <25%
Low grade
Male, age 69
Patient id: 3580
Staining: Medium
Intensity: Moderate
Quantity: >75%
H
TGM3
High grade
Male, age 71
Patient id: 4367
Staining: Not detected
Intensity: Negative
Quantity: None
Low grade
Male, age 57
Patient id: 3951
Staining: Not detected
Intensity: Negative
Quantity: None

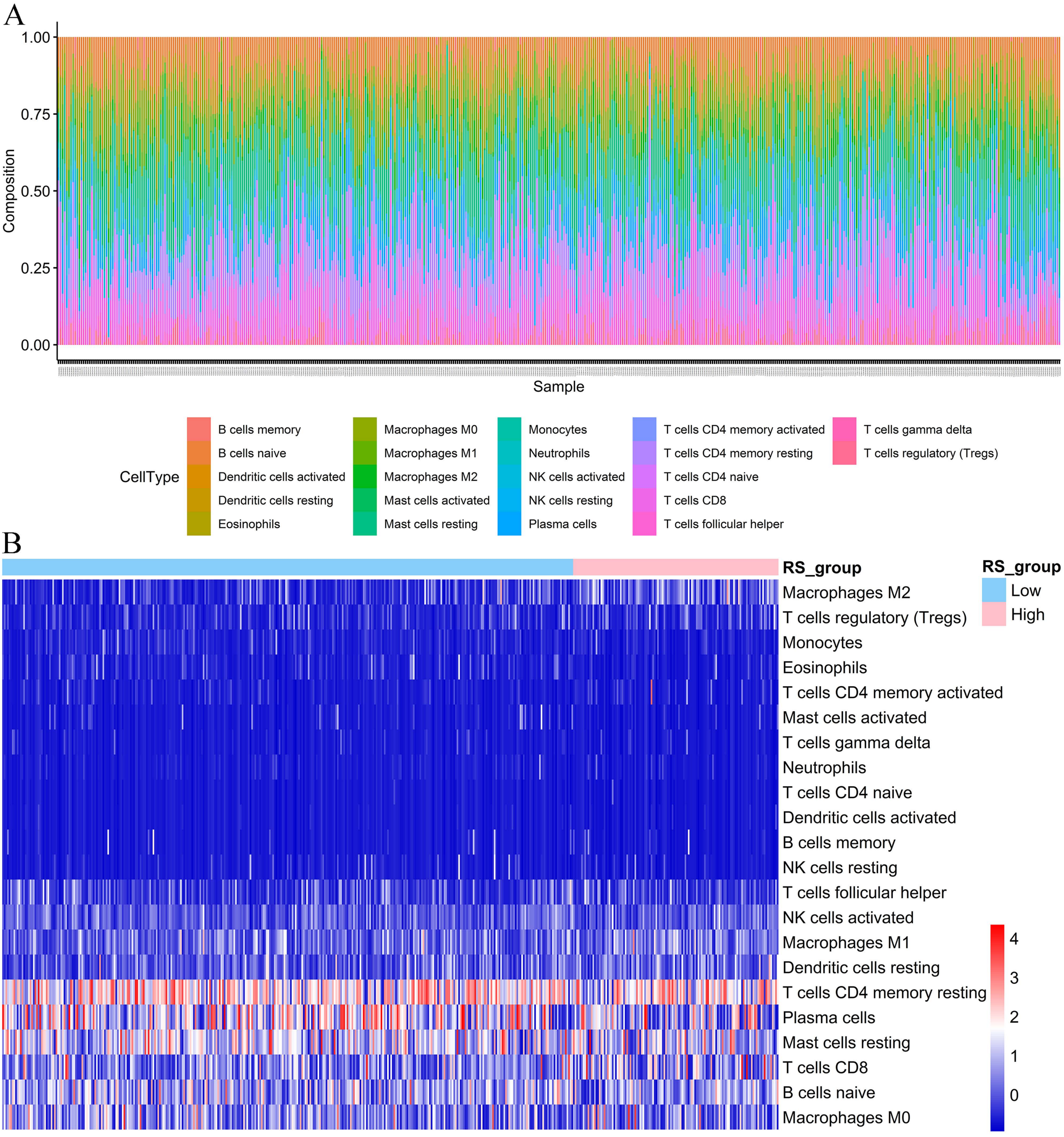
A
Composition
1.00
0.75
0.50
0.25
0.00
Sample
CellType
B cells memory
B cells naive
Dendritic cells activated
Dendritic cells resting
Eosinophils
Macrophages M0
Macrophages M1
Macrophages M2
Mast cells activated
Mast cells resting
Monocytes
Neutrophils
NK cells activated
NK cells resting
Plasma cells
T cells CD4 memory activated
T cells CD4 memory resting
T cells CD4 naive
T cells CD8
T cells follicular helper
T cells gamma delta
T cells regulatory (Tregs)
B
RS_group
Macrophages M2
T cells regulatory (Tregs)
Monocytes
Eosinophils
T cells CD4 memory activated
Mast cells activated
T cells gamma delta
Neutrophils
T cells CD4 naive
Dendritic cells activated
B cells memory
NK cells resting
T cells follicular helper
NK cells activated
Macrophages M1
Dendritic cells resting
T cells CD4 memory resting
Plasma cells
Mast cells resting
T cells CD8
B cells naive
Macrophages M0
RS_group
Low
High
4
3
2
1
0
